# “These are just finishing our medicines”: older persons’ perceptions and experiences of access to healthcare in public and private health facilities in Uganda

**DOI:** 10.1186/s12913-024-10741-6

**Published:** 2024-03-29

**Authors:** Stephen Ojiambo Wandera, Valerie Golaz, Betty Kwagala, James P. M. Ntozi, David Otundo Ayuku

**Affiliations:** 1 Department of Population Studies, School of Statistics and Planning, College of Business and Management Sciences, Makerere University, P. O. Box 7062, Kampala, Uganda; 2 Department of Mental Health and Behavioral Sciences and Mental Health, Moi University, Eldoret, Kenya; 3 Institut National d’Etudes Demographiques (INED), Paris, Aubervilliers France

**Keywords:** Drugs, Public health facility, Private health facility, Health system, Barriers, Facilitators, Elderly, Uganda, Sub-Saharan Africa

## Abstract

**Background:**

There is limited research on the experiences of access to medicines for non-communicable diseases (NCDs) in health facilities among older persons in Uganda. This paper explores the perspectives of older persons and healthcare providers concerning older persons’ access to essential medicines in Uganda.

**Methods:**

It is based on qualitative data from three districts of Hoima, Kiboga, and Busia in Uganda. Data collection methods included seven (07) focus group discussions (FGDs) and eighteen (18) in-depth interviews with older persons. Nine (9) key informant interviews with healthcare providers were conducted. Deductive and inductive thematic analysis (using Health Access Livelihood Framework) was used to analyze the barriers and facilitators of access to healthcare using QSR International NVivo software.

**Results:**

The key facilitators and barriers to access to healthcare included both health system and individual-level factors. The facilitators of access to essential medicines included family or social support, earning some income or Social Assistance Grants for Empowerment (SAGE) money, and knowing a healthcare provider at a health facility. The health system barriers included the unavailability of specialized personnel, equipment, and essential medicines for non-communicable diseases, frequent stock-outs, financial challenges, long waiting times, high costs for medicines for NCDs, and long distances to health facilities.

**Conclusion:**

Access to essential medicines for NCDs is a critical challenge for older persons in Uganda. The Ministry of Health should make essential drugs for NCDS to be readily available and train geriatricians to provide specialized healthcare for older persons to reduce health inequities in old age. Social support systems need to be strengthened to enable older persons to access healthcare.

## Background

Globally, the demographic transition associated with increment in longevity is expected to increase the proportion of adults age 50 and older. Currently, the proportion of older persons (those age 60) and older is 11% and is expected to increase to 20% by 2050. The absolute number of older people increased from 205 million in 1950 to 810 million in 201. Projections for 2022 and 2050 are one billion and 2 billion respectively, outnumbering children age 0–14 years [1, 2]. In sub-Saharan Africa (SSA), the absolute number of older persons (aged 60+) is 43 million, forming 5% of the population [3] and is projected to increase to 163 million (8.3% of the population) by 2050 [2]. In Uganda, the number of older persons has increased from 1.1 million in 2002 (4.5% of the population) to 1.3 million in 2010 [4] and shall increase to 5.5 million (5.7% of the population) by 2050 [2]. About 10% of the older persons were living alone by 2010 Uganda national household survey [5]. The average household size was 4.7 persons according to the Uganda Population and Housing Census of 2014 [6].

The World Health Organization (WHO) uses age 50 and older to define older persons in sub-Saharan African (SSA) countries due to lower life expectancy [7]. Several studies in SSA have used this definition [8–11]. On the other hand, the United Nations (UN) uses age 60 and older to define older persons [7, 12]. Likewise, the Ministry of Gender, Labour and Social Development (MoGLSD) in Uganda uses the same definition [4, 13]. Some reports on older people use this definition [1]. For this article, age 50 and older was used.

The prevalence of non-communicable diseases (NCDs) has been reported in some surveys in Uganda. Hypertension has been reported at 6.3% in the rural Uganda non-communicable disease (RUNCD) study [14]. A higher prevalence of hypertension (21%) has been reported among HIV patients [15]. A prevalence of hypertension of 21% has been reported in a sample of 611 people in a WHO STEPS tool study in Kasese [16]. Hypertension is the most reported NCD followed by obesity and diabetes in a scoping review for sub-Saharan Africa [17]. Among 2,382 older persons, about a quarter (23%) of the older persons reported an NCD in a 2010 national survey in Uganda [18]. Finally, in East Africa, 40% of deaths in 2015 were attributed to NCDs [19] and 53% were attributed to NCDs in eastern Uganda [20]. Therefore, NCDs are becoming an increasing health need in Uganda and are estimated at 26% [21].

In Uganda, the public healthcare system is hierarchically organized. At the top is the national referral hospitals: Mulago and Butabika. From Mulago national referral hospital, there are regional referral hospitals, health center (HC) from levels IV (district), III (sub-county), II (Parish) and I (village). Regional referral hospitals exist in each of the four regions of Uganda (central, eastern, western, and northern). At the lower levels, are health center IVs (district level) and IIIs (sub-county level). At the lowest levels namely parish and village are health center II and I, respectively [22–28].

Ageing is associated with several healthcare needs [29]. One of the critical needs of older persons is managing the worsening health outcomes [30] including non-communicable diseases (NCDs) and disabilities. NCDs increase the need for healthcare among older persons [31, 32]. This has been observed in Hong Kong [31], Singapore [33] and in rural South Africa [34]. However, in some low- and middle-income countries (LMICs), older persons with NCDs have limited access to healthcare. Examples include in India [35], China [36] and Hong Kong [31]. Generally, older persons face barriers of access to healthcare due to increased vulnerability and deprivation in old age [3, 30, 37, 38].

Studies on access to healthcare in general and access to essential medicines for NCDs and healthcare by older persons in Uganda are limited [39–42]. Available evidence on older people’s health have focused on HIV/AIDS [43–45]; caregiving roles of older persons [46–49] and various vulnerabilities [50, 51]. Studies which have addressed access to healthcare used quantitative secondary data and focused on patient level factors [32]. Others have focused on specific interest groups like the diabetics and not necessarily the older population [38, 52, 53]. Therefore, the aim of this paper was to explore perceptions and experiences about the health system and individual barriers and facilitators of access to healthcare among older persons in health facilities in Uganda. In addition, the perspectives of healthcare providers and community workers are explored using key informant interviews.

## Methods

### Study design, setting and sampling

This study used a cross-sectional study design. This qualitative study was a follow up to the secondary data analysis of the factors which predicted access to healthcare using a nationally representative household survey whose results are published elsewhere [32].

Three districts were purposively selected as study sites namely: Hoima, Kiboga and Busia, taking geographical and regional variations into consideration. Hoima was selected in western, Kiboga for central and Busia for eastern region.

Purposive sampling was used to select the study participants. Local leaders at the local council level guided the identification of older persons’ households. To recruit more participants, snow ball sampling was also used.

### Data sources

This paper primarily uses interviews from a qualitative study conducted in Busia, Kiboga and Hoima districts of Uganda in 2014 [54]. This study was part of a doctoral thesis for the the first author. Some of the survey data and findings are published in another paper [32]. In addition, preliminary interviews were conducted in 2012 in Hoima in a collaborative research framework on Poverty, Resource Accessibility and Spatial Mobility in East Africa [55]. Even though the data were collected in 2014, the findings are still relevant. First, there is limited evidence on the subject – experiences of older people with the public health system in Uganda. Some data which is yet to be published in other settings still points to the health system challenges for folder people. Recently, the Ministry of Health (MoH) in Uganda is in the process of developing a national healthcare strategy for older persons. Some of the findings have been used to inform this policy formulation process. Similarly, the Ministry of Gender, Labor and Social Development (MGLSD) is developing the national guidelines for mainstreaming ageing in Uganda.

### Data collection

The interviews with older persons included 18 In-depth interviews (IDIs) and seven focus group discussions (FGDs) collectively comprising 52 participants. We aimed to balance the gender of the different participants among the IDIs and the FGDs. Also, we aimed to do the same number of interviews in each district. The IDIs focused on exploring the perceptions and experiences of older persons about the barriers and facilitators of access to essential medicines for non-communicable diseases in health facilities. In addition, 9 key informant interviews (KIIs) were conducted with healthcare providers at public or private health facilities to facilitate triangulation of data. Healthcare providers were from Hoima regional referral hospital (Hoima), Bukomero health centre (HC) IV (Kiboga), Lumino HC III and Friends of Christ Revival Ministries (FOCREV) clinic (Busia district). The KIIs focused on the barriers and facilitators of access to essential medicines among older persons in Uganda. All the interviews were audio recorded. Entry into community was sought through village local council (LCs) chairpersons, older person’s associations in Hoima and Busia, and SAGE coordinators in Kiboga district and health facility in charges.

### Ethical considerations

The study was approved by the Research Ethics Committee / Institutional Review Board (IRB) of the Uganda National Council of Science and Technology (UNCST) (SS 3198). The multi-disciplinary study on Poverty, resource accessibility and spatial mobility in East Africa (MPRAM) research programme was also approved by the IRB of the UNCST (SS 2726).

All experiments / research processes were performed in accordance with relevant guidelines and regulations (such as the Declaration of Helsinki).

All the respondents gave their written and informed consent to participate in the study. During the informed consent process, we provided assurance of confidentiality, participation on voluntary basis, freedom to withdraw or to decline and to answer any question without negative consequences. Finally, during the reporting and publication phase, we anonymized the interviews to ensure confidentiality of the interviewees.

### Inclusion and exclusion criteria

The inclusion criteria were being age 50 years and older, being in the right mind and the ability to comprehend. In addition, we aimed to interview older persons who were not ill by the time of the interview. Also, eligibility criteria included older persons who were in their right mind and could comprehend and were not ill by the time of the interview.

### Data analysis

All recorded interviews were transcribed verbatim. All transcriptions were checked to ensure accuracy in transcription and translation from local languages (Lunyoro, Lusamia and Luganda for Hoima, Busia and Kiboga respectively) to English. Transcriptions were later, imported into QSR International NVivo software (version 9) for thematic and / or framework analysis [56].

Both deductive and inductive thematic analysis were used in the coding exercise [57]. Themes were developed following the Health Access Livelihood Framework (HALF model) related to access to healthcare [58]. The HALF model describes five dimensions of access to healthcare: availability, affordability, accessibility, adequacy and acceptability and five dimensions of livelihood assets [58]. The inductive thematic analysis involved adding themes or codes that were emerging from the transcriptions during the coding exercise.

## Results

This section presents the facilitators and barriers of access to healthcare among older persons. Perceptions and experiences are explored from the older persons and healthcare providers. We start with the facilitators and end with the barriers.

### Facilitators of access to healthcare among older people

The facilitators of access to healthcare included availability of public health facilities, social support, support from NGOs, access to financial resources, transportation, access to village health teams, and having a healthcare provider who is a relative.

#### Availability of free services in public health facilities

Availability of public health facilities which are expected to provide free medical services was highlighted as a motivation for visiting health facilities. In addition, non-communicable diseases create demand for healthcare. Private health facilities are not easily afforded by older people. They prefer to visit public health facilities:

… we go there when we fail to get money to take us to private health facilities…When a person is very cold and you make a fire, you do not have to invite him. He will bring himself. We go there because we are sick… You wonder whether they will give you medicine or not… **[Male FGD, Hoima]**

Putting a **special clinic day for older persons** was considered as a facility for access to healthcare in Hoima. Older persons continued to access healthcare on all days. However, a special day was designated to provide extra care for older persons. After some time, this was removed. However, this was not standard practice across all the three districts. It was the best practice in Hoima only. This was being at the regional referral hospital. Conversely, older persons complained that “sickness does not wait for you on a special day”. As much they were happy with a special or clinic day for older persons, they expressed concerns about its effectiveness.

#### Family and social support

Family support includes the support of spouses, relatives, brothers and sisters, adult children, and grandchildren. The form of support could be material, financial, or physical/caretaking. Older persons with highly educated working children, and those with children working abroad tend to receive significant support. Relatives play a major role in patient attendance and interacting with health personnel. In kind /material support for instance includes purchase of food, eyeglasses, and transfer to better health facilities around the city. Older persons with relatives working in health facilities favour them by helping them to “jump” the queue. Many older persons receive support from family members in form of means of transportation to health facilities and payment of medical bills.

Some of us have educated our children like you [refers to moderator]. That is a real bank. When you educate a child and he gets a job, that is a bank **[Male FGD, Hoima]**

However, family, or social support dynamics are changing. Some older persons reported that family support is limited and is not reliable. Some older persons with relatives who can transport them to hospital and pay the bills are very few. This was stated in an FGD in Kiboga. Children whose resources are meagre are unable even though they would be willing to support their parents because they also must cater for their own children/families. Other children may not even help when they are able to. This was emphasized in one FGD:

… The health of old people is very poor. Some have children who do not care for them. Even those who have no children have nobody to care for them… **[Male FGD, Hoima]**

#### Support from non-governmental organizations (NGOs)

Some NGOs in Busia provided support to older persons including Compassion International. Compassion International in Busia provided some support to older persons as caregivers of children, when they have chronic illnesses such as TB, HIV, and cancer. In Hoima, the Uganda Reach the Aged Association (URAA), World Vision, Sans Frontiers, Sight Savers International, Infectious Diseases Institute (IDI) and Little Hospice Hoima provided health support. In Kiboga, Stearkey (based in Ntinda), provided support to older people.

When they have chronic illnesses, we intervene …like TB, we support like HIV groups for HIV positive…. umm they are mostly old people **[Community Worker, Busia]**

The URAA trained home-based care givers in Hoima who offered basic treatment to older persons from their homes. However, the program ceased due to lack of funding.

There was an NGO helping the elderly. They trained the home-based caregivers. They could bring drugs for the elderly… It was Uganda Reach the Aged Association, URAA… But they ran short of money and all that… **[Female FGD, Hoima]**

Little Hospice takes care of severe chronic illness such as stroke or cancer for older persons who are on HIV treatment. Infectious Diseases Institute (IDI) was supporting HIV ART in Hoima regional referral hospital where older persons were beneficiaries. Sight Savers and Sans Frontiers provide eye care, provision of eyeglasses inclusive and hearing aids respectively. STEARKEY from Ntinda, Kampala, Uganda, also supports older persons with hearing aids in Kiboga.

…with sight, we have got the Sight Savers… at least that one yes… umm sight savers and also hearing umm at least there there people Sans Frontiers, who usually come around. and then after checking… I think they give out spectacles… free free… Sans Frontiers they they cross check they check for the the the ears impairment **[Traditional Minister, Bunyoro Kingdom, Hoima]**

Some older people with disability received some transportation assistance from the National Union of Disabled Persons Uganda (NUDIPU).

#### Financial resources

Having financial resources / money helped older persons to access private health facilities, where the services / handling was perceived to be better than in public health facilities. Generally, poor older persons use public health facilities while the rich or the middle class use private health facilities. Financial resources are essential in purchasing essential medicines or drugs for NCDs and paying for extra charges at public facilities. A community worker noted:

…the haves go to private (facilities), the have-nots they stay in queues waiting for medicines [public health facilities] … and the have-nots, they have no choice they have to wait in the queues… when you have your money, why should you wait? **[Community worker, Hoima]**

Older persons in Kiboga, who received the monthly Social Assistance Grants for Empowerment (SAGE) funds, were able to use some of the resources to access healthcare. Older persons in Busia and Hoima were not receiving the SAGE grant by that time. These two districts were not yet included on the beneficiary list. A community worker in Kiboga asserted:

…the biggest challenge for the elderly is that they have been lacking money… now when you give them an opportunity to have cash [SAGE grant] … even if he falls sick, he still is able to … buy drugs **[Community worker, Bukomero, Kiboga]**

#### Availability of transport to access healthcare

Availability of transport to visit the health facilities was a key facilitator. The means of transport are usually provided by older persons’ relatives, children and sometimes, NGOs. A community worker in Hoima noted:

… those who have relatives who are able… to move them on boda bodas or even bicycles… or even vehicles…there are some rich people **[Community worker, Hoima]**

Some NGOs like World Vision and Bukomero Development Foundation in Kiboga provided transport to older persons in Kiboga to access better treatment in Kampala. A community worker in Kiboga stated that:

There are those older people especially those that are suffering from HIV and AIDS, … we have been able to give them transport to go for medication may be in Mulago … to be able to access better treatment from Kampala **[Community worker, Kiboga]**

### Access to village health teams (VHTs)

The community health system in Uganda includes village health teams (VHTS), community health workers (CHWs), health assistants (HAs), expert clients and the AIDS community volunteers (ACVs). VHTs and CHWs identify, refer to health facilities, and give health education to older persons. This has helped TB and HIV patients to access treatment. This was the major outreach strategy in Lumino HC 3 (Busia) and Mparangasi HC 3 (Hoima district). Some older persons visit health facilities after such referrals. A healthcare provider in Busia reported:

… we have some patients in the village who are chronically sick…they do not go to hospitals … so the VHTs or the CHWs identifies them… visits them and they have referral notes, they do refer them to us… so some of them come … **[Healthcare provider, Busia]**

Those who do not turn up are followed by medical staff from the health facility, which is an outreach strategy to the community. The VHTs majorly deal with malaria treatment among children and pregnant women. One KII in Busia emphasized:

…at times those who are referred, and they do not come… So, we send our people now… we even send these nurses… to go and visit these patients in the village…. it is more active on TB and the ART clinics **[Healthcare provider, Busia]**

However, in Kiboga, some participants noted that VHTs focus on children and not older persons:

…We also have VHTs… they are dealing with treatment of children… specifically malaria but they do sensitization and mobilization… they are supposed to tell the old people, … to even advocate for them **[Healthcare provider, Kiboga]**

#### Knowing a healthcare provider at a health facility was a great motivator

Access to healthcare was much easier when an older person knew a healthcare provider - as a relative or a son or daughter of a friend. In such cases, he or she is helped to jump the queue. He or she is removed from the line and treated first irrespective of whether he or she came earlier or later than other patients. In some cases, relatives who can talk well to providers also help them jump the queue. In some other instances, providers offer direct assistance to older persons. An older woman in Busia asserted:

No, for me they make me jump the queue… Yes, there are those I know… There is Sam (Pesudo name), he just picks me… I don’t know the work he does but he comes that ‘elder come they work on you and you go’… They can complain about it when am not there…They finish complaining and for me, I am gone. It is upon them as a person who backbites you after you had left, can you hear? **[Older woman, age about 70, Busia]**

In Hoima, mention of such assistance also came up in the male FGD:

If you know some member of staff, someone’s child working there… I know the doctor. The relationship at that level. Someone can leave the health facility in Kasomoro and come to Booma because there is the child of the aunt working there **[Male FGD, Hoima]**

### Barriers of access to healthcare

The barriers of access to healthcare for older people were numerous. They ranged from lack of essential medicines to ageism against older people, absence of geriatricians, treatment adherence issues, accessibility, affordability challenges, and acceptability issues. These are described as follows:

#### Availability of essential medicines for non-communicable diseases is a critical gap

This was the most frequently discussed theme in al the interviews. Both older persons and healthcare providers acknowledged unavailability or frequent stockouts of essential drugs for non-communicable diseases (NCDs) for older people in public health facilities. Public health facilities normally refer older people to private clinics to buy medicines, which is a huge barrier for them. Many older persons reported frequent drug stockouts at public health facilities due to large numbers of patients^1^. An older man in Busia witnessed a situation where the medicines, which were received at a health centre III in Busia district, only included septrin and paracetamol. In Busia, older persons reported challenges with the distribution of drugs:

When we go to VHTs, they tell us the drugs are for the young children; that we should go to the health facility to get ours. But when we go to health centres, we do not find the drugs there **[Male FGD, Hoima district]**

There was a belief that public health facilities receive medicines that do not match “older people’s” sicknesses (refers to NCDs) in health centre IIIs. According to one of the health providers in a health centre III, it is rare to find drugs for hypertension, diabetes, and typhoid at health centre IIIs^2^ and lower levels.

Some older persons in Busia suspected **drug pilferage***by health providers - that the drugs are then sold in* health providers’ private clinics. However, health providers in Busia attributed unavailability of some drugs to frequent stock outs because of the *government push system*, which predetermines which drugs are sent to HC III and II and the heavy client flow in public health facilities. In addition, absence of storage facilities for insulin explained the shortage of insulin for diabetes in one of the regional referral hospitals in Hoima^3^.

One of the health providers in a health centre III explained that health centres IIIs receive medicines from district hospitals because of the **government “push system”** (KII, Busia). Although medicines or drugs in public facilities are supposed to be free, patients are sometimes asked to pay some money to the dispensers to help them purchase the drugs which are not available from an outside private pharmacy. Participants perceived this to be a form of extortion as noted:

Dispensers tell you that the drug is out of stock. Once you give him the money, he just pockets it and then picks up the medicine and gives it to you…. assuming that he has just bought it from outside the clinic **[Community worker, Hoima]**

#### Absence of specialized health personnel including geriatricians

The second most pressing barrier was concerned with healthcare providers especially geriatricians. The unavailability of health personnel trained in geriatrics and gerontology makes it difficult to adequately addressing older persons’ health problems. A key informant referring to lower-level health facilities alluded to the need to have trained doctors in Hoima:

The health facilities exist but health providers cannot manage the complications of the diseases of the elderly… because most of these health centers are managed by nurses. A man may have problems associated with hypertension, or the heart; such health centers cannot handle even if they are near **[Community worker, Hoima]**

#### Ageism against older people by young healthcare providers

One of the key factors was ageist and negative attitudes against older people by young health providers in public health facilities. Older people in Busia reported *ageism* perpetrated by young health providers. Older persons were reprimanded for trying to access medicines instead of giving space and priority to younger persons. Female FGD participants in Busia cited a health provider’s statement as follows:

‘These (older persons) are just finishing our medicines; don’t they have grandchildren?’ With such observations from health providers, … we get tired of going to the hospital… They tell us that we finish medicines for our grandchildren…. Now they say we are useless to people **[Female FGD, Busia district]**

Poor handling of older people by healthcare providers was attributed to both the overwhelming workload and the intentional mistreatment by young nurses. The most vulnerable older persons are those that seek for care on their own (alone).

#### Affordability and financial challenges

Poverty or financial barriers was the fourth most reported problem in accessing healthcare. Older persons found challenges in paying for medical bills, drugs, transport costs and extra charges. In addition, specialized tests and services were unaffordable. Specialist services included eye care, surgical operations for appendicitis, tubal blockage, and hernia. Specialized tests like X-ray and CT scans were costly for older persons. An older woman in Busia, who was blind due to cataracts, had not gone to Mbale hospital, where she was referred because of money problems. In Hoima, older women in an FGD had this to say:

…You do not have money to buy medicine every time you go to the health facility. This disease … high blood pressure does not get healed. It is like AIDS or even more than AIDS… **[Female FGD, Hoima district]**

#### Referrals and prescription challenges

Most older people find it difficult to follow referrals to buy medicines or drugs from private clinics because of financial and literacy barriers. In addition, they were challenged to adhere to treatment prescriptions. On the other hand, private health facilities stock drugs for NCDs but at a fee, that is often very expensive for many older persons. A health provider in Busia described referral process for older persons as a “mountain climbing” experience:

Due to financial limitations, in some cases, older persons purchased incomplete dosage depending on their wallets^4^: An older person in Hoima district noted the following:

Health providers can prescribe a full dose that may cost 9000 shillings, but you cannot raise it at that time. You buy half dose for 4500 shillings which you can afford at that time **[Older man, age 70+, Hoima]**

Referrals to buy essential drugs for NCDs from private facilities, which are not available in public health facilities, were unaffordable to older persons. Besides, private clinics were found to be expensive.

#### Shortage of specialized equipment to screen and test for NCDs

Many public health facilities lacked some specialized equipment and health services for screening and diagnosing NCDs. For example, the computerised tomography scan (CT scan) and ultrasound scans. By the time of the interview (during the M-PRAM project in 2012), Hoima regional referral hospital (RRH) neither had a CT scan machine, nor an ultrasound scan. In private clinics, CT and ultrasound scans cost between 30,000 and 50,000 UGX, which is expensive for many older persons. In addition, X-ray cost 15,000 UGX even in Hoima RRH by the time of the study. Concerning health services, a key informant noted:

… Western medicine is not affordable. You must go to a clinic, pay money for checkup, and the medicine, because in the government facilities, the CT scan services are not available **[Community worker, Hoima]**

#### Accessibility challenges

Long distance to health facilities limited older persons’ access to healthcare. This challenge is compounded by physical disabilities and caretakers’ reluctance to take older people to health facilities. Consequently, older persons fail to keep appointments with health providers for instance, crucial ones like diabetes management.

In addition, a lack of means of transport was another barrier to accessing healthcare or visiting distant health facilities in the event of referral. Public transport is not readily accessible in remote areas. Ambulances in public health facilities are non-functional due to mechanical challenges or fuel shortages.

Transport costs are a serious challenge to older persons. Some older people do not have money to pay for transportation to a health facility. Means of transportation used ranged from a bicycle, boda boda (motorcycle) which is the most common, to a vehicle.

Physical disabilities among older people usually make it difficult to access health facilities. They may be unable to walk on their own when the illness is severe. Sometimes, they are unable to ride bicycles by themselves or they don’t even own one. They may not have funds to pay a boda-boda (motorcycle). Older people with disability could not walk to health facilities on their own. They depend on caretakers.

…with age, they come complaining … of painful legs, painful lower limbs …by the time they become real disabled, you can’t see them in hospital… they … stay home… they may fail to bring them to hospital **[Health provider, Hoima hospital]**

Social stigma also prevented many disabled older persons from being brought by their care takers to health facilities. A community worker narrated the ordeal:

… a totally blind HIV positive older person died alone in the house in Hoima and was buried on 26th July 2012 **[Community worker, Hoima]**

#### Adequacy and quality considerations in public health facilities

Some hospitals organized health services on specific days which made it difficult for older people to access care on the non-scheduled days. In addition, irregular or short working hours were reported as critical impediments to access to and utilization of health services. For example, older patients complained about late opening time (about 10 am) and early closing time (about 4 pm) except for special clinic days for children and expectant mothers.

In addition, mixing older persons with younger patients (especially children and women) who are stronger was reported as another barrier. Older persons reported that they had no strength to queue for services for long hours. This is a major impediment to their access to services in public health facilities, which are usually crowded with sick children and their mothers. Owing to long waiting hours in queues some older persons reported that fail to retain urine and as a result, they get ashamed in public. Apart from the heavy client flow in public facilities long queues and waiting time were attributed to staff shortages. For example, the doctor to patient ratio in Hoima regional referral hospital was about 1:100.

…One doctor can see over 100 patients… so they have to keep in the line, and when they come, you have to send them to the lab for a random blood sugar test… by the time she comes again she has to queue again … that becomes a problem **[Health provider, Hoima]**

#### Acceptability problems

Personal stigma limited-service acceptability by older persons. Some older persons expressed self-directed stigma - a feeling that they would not be cared for by health providers when they visited health facilities. In addition, some older persons reported not accessing care because of lack of presentable clothes to wear for health facility visits. Some who had bad experiences are health facilities indicated that they preferred death to discrimination and disgrace at public health facilities. According to them, death would mean rest from suffering. A community worker who interacts with older persons made the following observation.

…some older persons have given up accessing services at public health facilities. Even if you tell them to go to hospital, they say ‘no, don’t take me there’, because they know, the moment they are taken to hospital, they are mistreated… they are as rejects. Old people say ‘please leave me alone if am to die, let me just die peacefully at home other than being tossed about **[Community worker, Hoima]**

Traditional and religious beliefs in some communities promoted herbal medicine and discouraged western medicine. In Hoima district, some adherents of the religious sect called “Wobusobozi” [meaning he is able] led by “Bisaaka” do not believe in modern or western medicine:

The fear of Bishaaka himself he has made them believe that he knows whatever they do … or what they think. So, his word is final… health wise, it’s because he did not believe in modern medicine but when he saw that that it was putting him on a clash with government, he has changed **[Community worker, Hoima]**

## Discussion

The aim of this paper was to investigate the barriers and facilitators of access to healthcare among older persons in Uganda at both the health system and individual levels. Our findings indicated that barriers outweighed facilitators. The barriers and facilitators of access to healthcare tended to overlap. Key facilitators included availability of free services, social support, financial affordability, transportation, village health teams and knowing a healthcare provider. On the other hand, the barriers included unavailability of essential medicines for NCDs, specialized personnel and equipment, ageism among healthcare providers, financial challenges, poor quality and acceptability problems of public health facilities. The facilitators and barriers are either health system or patient factors.

### A challenging health system

A series of challenges stemming from the health system itself, that is acknowledged by community workers, health sector workers as well as by the older persons, stem out from this study. From the health system side, absence of geriatricians, unavailability of essential medicines for NCDs, ageism, affordability limitations and acceptability challenges were critical gaps in Uganda.

**The unavailability of essential drugs for NCDs** in lower public health facilities (health center IIIs and lower) was a major barrier of access to healthcare for older people. The perceptions of the older persons and community workers generally were that “healthcare providers sell drugs through their clinics”. Conversely, healthcare providers explained that the government uses a push system which provides basic medicines to lower health facilities. The government pushes medical supplies and medicines to lower health facilities (health centers I to III). That is, “larger public health facilities express significantly greater facility-level autonomy in drug ordering compared with smaller facilities, which indicates ongoing changes in the Ugandan medical supply chain to a hybrid ‘push-pull’ system” [59]. Larger health facilities include national, regional and district health facilities. These make orders for their medicines depending on need of the people served. There is tension about these perceptions from older people and the explanation by healthcare providers. Some studies have reported this challenge, absence of essential medicines for NCDs in the Ugandan heath system [23, 38–41, 60–62]. The same shortage of supply of essential medicines for NCDs is reported in Kenya, Cameroon and the DRC [62, 63].

In Uganda, the Ministry of Health (MoH) adopted the dual “pull and push” system in 2010 in the delivery of essential medicines and health supplies (EMHS) [42, 64]. The pull system was maintained for HC IVs and hospitals while the push system was introduced for rural and hard to reach health facilities including HC III and IIs. Lower-level health facilities no longer request for drugs based on the diseases’ burden and the population served [42, 65–67]. HC IIIs do not request for the drugs they need except for tuberculosis (TB) and antiretroviral drugs [42, 64]. In lower public health facilities (HC I-III), older persons are given drugs that are available and referred with prescriptions to buy drugs from private clinics for those that are not available^5^. The implication is the unavailability of essential medicines for NCDs in HC IIIs and IIs, which are accessible to older persons, because they no longer request for drugs /medicines based on the diseases’ burden and the population served [65–67]. Older people who visit public health facilities cannot obtain treatment according to prescriptions in lower-level health facilities. Referrals to purchase medicines from private facilities is a nightmare to them. They end up with no treatment or accessing less than the required dosage. This has negative implications on older persons’ health outcomes including disease progression and drug resistance [40, 63]. NCDs require long term adherence to treatment regimes. This is interrupted when availability of medicines and supplies ins interrupted [40]. In the absence of essential medicines, some older persons resort to herbal medicine. However, their preference would be essential medicines from health facilities.

**Generally, there is an acute scarcity of skilled healthcare providers** for handling NCDs among older people in Uganda, particularly geriatricians and gerontologists. This was a serious concern among healthcare providers, older persons, and other community workers. Older people also agreed with the situation as they indicated that most times, they were handled by nurses and clinical officers and few doctors. Older persons are handled by clinicians, physicians and nurses who sometimes have no clue about handling multi-morbidity and polypharmacy among older people. This was an issue of consensus across all interview types. Other studies also report the absence of skilled providers as major barrier [60, 61]. Chronic non-communicable diseases create greater need for healthcare [31], in countries such as Hong Kong [31], Singapore [33], and in rural South Africa [34]. Older persons still report limited access to healthcare [35].

**Ageism in healthcare delivery** was reported among some young healthcare providers especially nurses. Sometimes, lower-level healthcare providers do not have training to handle NCDs and therefore, end up manifesting ageist attitudes to older people in their delivery of healthcare. Although some older people reported ageist attitudes by some healthcare providers, others reported good experiences with some especially when they knew them. The experiences vary from one individual to another. Ageism is reported as a critical impediment for older persons’ access to healthcare [29].

Health services were considered **inadequate and of poor quality** by both older persons and healthcare providers. Adequacy relates to how healthcare is organized and whether that meets patients’ expectations [53]. For example, opening hours and waiting times. In addition, it covers hygiene, and quality of care [58, 68]. Irregular working hours have been reported as major barriers to access to healthcare in Uganda [58, 60, 68]. Long waiting times are documented as critical impediments to access to and utilization of health services in Uganda [60, 68]. Generally, public health facilities were perceived as those which provide poor quality services [63]. Private health services were perceived as those with better quality but not affordable to older people.

Finally, **acceptability challenges** were reported in the health system. Acceptability refers to cultural access [69] or socio-cultural access [68]. It relates to providers’ and patients’ attitudes, beliefs and expectations of each other [69]. It also includes patients’ perceived quality of care [61, 68] and satisfaction with care [68]. Obrist et al. (2007) argues that for effective healthcare access, the patients must feel welcome, cared for by service providers and must trust in the competence and personality of the healthcare providers” (Obrist et al., 2007). The consensus among older people was that public health facilities were not acceptable to them. The essential medicines were lacking, specialized equipment for diagnosis were either absent or if present, very costly for them and the providers were nurses who did not understand how to handle or manage NCDs. However, a special clinic day for older persons was a great facilitator. It was an effective intervention for older people.

### Individual challenges

**Physical disability** and mobility limitations was a significant barrier of access to healthcare. Disability reduced physical accessibility to health facilities. Accessibility focuses on the geographic distance and travel time between users’ homes and the nearest health facilities (Obrist et al., 2007; Peters et al., 2008). It also includes (un)availability of public transport and ownership of a means of transport e.g. bicycle or motorcycle [58]. This finding was reported in the quantitative data as well [32]. The health access livelihood framework (HALF) posits that vulnerability context affects access to healthcare [58]. Disabled older persons are unable to move to health facilities on their own. They need a means of transport and a caretaker to assist them move to a health facility. Some care takers are either unwilling or lack the means to transport older persons to health facilities.

**Family support** and its absence have been mentioned as both a facilitator and a barrier when absent [41]. This is important in terms of financial means, support for transport but also care, responsibility. Children are call upon by older parents to take them through the difficulties they face – here specifically health issues, by swiftly making the right decisions. Yet, children are sometimes far away and not in a position to provide the expected support. Availability and absence of financial means affects affordability of healthcare for older people.

**Affordability** of specialized healthcare for NCDs is a major limitation for older persons. Most older persons lack health insurance in the absence of a national health insurance scheme in Uganda. Paying out of the pocket medical expenses is unattainable since most older persons do not have pensions in old age [13, 40, 70]. Olde age poverty is a big problem in Uganda [50]. In addition, most NCDs treatment is very costly even though it is readily available in private health facilities, but not public ones. Older persons end up leaving hospitals without medicines or with half dosages or resorting to non-medical alternatives [40, 63]. Referral to purchase medicines in private facilities and pharmacies is a big problem [40]. In Uganda, health insurance schemes do not cover some or most NCDs [41]. Affordability refers to financial access [69] or financial accessibility [68]. The costs of healthcare services are expected to fit the clients’ resources or income and willingness and ability to pay (McIntyre et al., 2009; Obrist et al., 2007; Peters et al., 2008). Affordability relates to direct costs e.g. user fees, payment for drugs; indirect costs e.g. in terms of transport costs, lost time and income and other “unofficial charges” such as paying bribes (Obrist et al., 2007). Some study indicated the acute affordability challenge of medicines for NCDs in Congo DRC and Cameroon [62] and Kenya [63]. Finally, access to healthcare among older persons is affected by the individual’s confidence in the health system, the ability of the patient to afford care, the health system’s capacity to respond to individual needs with respect and dignity.

## Strength and limitations

This study was a follow up of a quantitative study whose results have already been published [32]. Here, the triangulation of qualitative data collection methods (FGDs, IDIs and KIIs) improves the validity and reliability of the data. The integration of healthcare providers’ perspectives with those of the older persons, gives a consistent picture to the barriers and facilitators of access to healthcare among older persons in Uganda.

It would have been possible to go further in the analysis of factors and barriers in access to healthcare with follow up interviews and observation sessions of situations when older people access healthcare or would want to and of service delivery in healthcare facilities. This would call for participant observations, within villages and around some health facilities which handle older persons which wasn’t planed for in our overall project using an explanatory mixed methods research design. It however remains the major limitation of this qualitative study.

Finally, using the data when the interviews were conducted in 2012 and 2014 needs to be acknowledged. The timing is quite long. One would argue that times and seasons have changed regarding access to healthcare! However, the firs author has been involved in the policy formulation process for developing the national healthcare strategy for older persons in Uganda by the World Health Organization and the Ministry of Health. The findings are still relevant and the new evidence from the MOH officials tallies with some of the reported findings.

## Conclusions & recommendations

Older persons face immense health system and patient level barriers when accessing healthcare in Uganda. Older persons have greater heath needs because of NCDs and functional limitations. However, the health system in Uganda is still unresponsive and insensitive to the health needs of older persons.

Major health system barriers include inadequate supply of essential drugs for NCDs, absence of geriatricians among healthcare personnel, low acceptability by younger healthcare providers, long waiting times, long queues, unaffordability of certain specialized services, inaccessibility of some facilities, and discrimination in the health services’ delivery.

At the patient level, there are social inequalities in access to healthcare among older people. The major barriers are financial, transport challenges, physical disability, and multi-morbidity because of NCDs.

Key recommendations to improve access to healthcare for older persons include the following: First, the health system needs strengthening to be able to respond to the health needs of older people in Uganda. Second, the tracking of the supply of essential medicines for NCDs for lower-level health facilities is critical. Third, training of geriatricians for the health system and social gerontologists, would be key interventions urgently needed to address this gap. Fourth, a national health insurance scheme to cover all vulnerable groups including older persons is warranted. Finally, developing a national policy and health strategy for addressing access to healthcare by older persons is urgently needed.

## Data Availability

The datasets used and/or analysed during the current study are available from the corresponding author on reasonable request.

## Footnotes

1 IDI 15, older man age 70, Busia district. He visited HC thrice and missed drugs once when he was referred to buy and did not have the money. He also stated that the prescription was written in English which he could not understand.

2 KII_13, provider at HC III, Busia.

3 KII_05, Hoima regional referral hospital.

4 IDI 01, older man age 64, Hoima. Had high blood pressure but was better off economically.

5 IDI 13, older man age 56, Busia district. He also reported that he was once injected with an expired drug in one of the clinics by a nurse, who didn’t check the label/expiry date of the drug.

## References

[1] UNFPA HAI (2012). Ageing in the twenty-First Century: a celebration and a challenge.

[2] UN, World Population Prospects. The 2010 Revision New York: United Nations, Department of Economic and Social Affairs, Population Division 2013. Available from: http://esa.un.org/unpd/wpp/unpp/p2k0data.asp.

[3] Aboderin I, Ferreira M. Linking ageing to Development Agendas in Sub-saharan Africa: challenges and approaches Population Ageing. 2008;1:51–73. 10.1007/s12062-009-9002-8 PMC6485422 31031871

[4] UBOS. Uganda National Household Survey 2009–2010. Socio-economic module. Abridged report. Kampala, Uganda: Uganda Bureau of Statistics; 2010 November.

[5] Wandera SO, Ddumba I, Akinyemi JO, Adedini SA, Odimegwu C (2017). Living alone among older persons in Uganda: Prevalence and Associated factors. Ageing Int.

[6] UBOS (2014). Uganda Population and Housing Census 2014.

[7] WHO. Definition of an older or elderly person: Proposed Working Definition of an Older Person in Africa for the MDS Project Geneva, Switzerland: World Health Organization. ; 2015. Available from: http://www.who.int/healthinfo/survey/ageingdefnolder/en/.

[8] Minh HV, Byass P, Chuc NTK, Wall S. Patterns of health status and quality of life among older people in rural Viet Nam. 2010. 2010;3(Supplementary 2):64– 9. 10.3402/gha.v3i0.2124 PMC2957159 20959877

[9] Mwanyangala MA, Mayombana C, Urassa H, Charles J, Mahutanga C, Abdullah S (2010). Health status and quality of life among older adults in rural Tanzania. Global Health Action.

[10] Ng N, Hakimi M, Byass P, Wilopo S, Wall S. Health and quality of life among older rural people in Purworejo District, Indonesia. Global Health Action 2010(Supplement 2). 10.3402/gha.v3i0.2125 PMC2957148 20959875

[11] Ng N, Minh HV, Juvekar S, Razzaque A, Bich TH, Kanungsukkasem U et al. Using the INDEPTH HDSS to build capacity for chronic non-communicable disease risk factor surveillance in low and middle income countries. Global Health Action. 2009;2(1984). 10.3402/gha.v2i0.1984 PMC2785135 20027262

[12] Africa Health S, AU (2007). 2007–2015.

[13] MoGLSD. National Policy for Older Persons (2009). Ageing with security and Dignity Kampala.

[14] Siddharthan T, Kalyesubula R, Morgan B, Ermer T, Rabin TL, Kayongo A (2021). The rural Uganda non-communicable disease (RUNCD) study: prevalence and risk factors of self-reported NCDs from a cross sectional survey. BMC Public Health.

[15] Kansiime S, Mwesigire D, Mugerwa H (2019). Prevalence of non-communicable diseases among HIV positive patients on antiretroviral therapy at joint clinical research centre, Lubowa, Uganda. PLoS ONE.

[16] Mondo CK, Otim MA, Akol G, Musoke R, Orem J (2013). The prevalence and distribution of non-communicable diseases and their risk factors in Kasese district, Uganda. Cardiovasc J Afr.

[17] Mudie K, Jin MM, Tan, Kendall L, Addo J, Dos-Santos-Silva I (2019). Non-communicable diseases in sub-saharan Africa: a scoping review of large cohort studies. J Glob Health.

[18] Wandera SO, Kwagala B, Ntozi J (2015). Prevalence and risk factors for self-reported non-communicable diseases among older ugandans: a cross-sectional study. Glob Health Action.

[19] Kraef C, Juma PA, Mucumbitsi J, Ramaiya K, Ndikumwenayo F, Kallestrup P et al. Fighting non-communicable diseases in East Africa: assessing progress and identifying the next steps. BMJ Glob Health. 2020;5(11). 10.1136/bmjgh-2020-003325 PMC7662421 33184064

[20] Natukwatsa D, Wosu AC, Ndyomugyenyi DB, Waibi M, Kajungu D (2021). An assessment of non-communicable disease mortality among adults in Eastern Uganda, 2010–2016. PLoS ONE.

[21] Meghani A, Ssemugabo C, Pariyo G, Hyder AA, Rutebemberwa E, Gibson DG (2021). Curbing the rise of noncommunicable diseases in Uganda: perspectives of policy actors. Glob Health Sci Pract.

[22] Ssensamba JT, Nakafeero M, Musana H, Amollo M, Ssennyonjo A, Kiwanuka SN (2022). Primary care provider notions on instituting community-based geriatric support in Uganda. BMC Geriatr.

[23] Bakeera S, Petzold M, Pariyo G, Galea S, Tomson G, Wamala S (2010). Community social capital and the use of health care services in Uganda. J Public Health Epidemiol.

[24] Bakeera S, Wamala S, Galea S, State A, Peterson S, Pariyo G (2009). Community perceptions and factors influencing utilization of health services in Uganda. Int J Equity Health.

[25] Odaga J (2004). Health inequity in Uganda: the role of financial and non-financial barriers. Health Policy Dev.

[26] Pariyo G, Ekirapa-Kiracho E, Okui O, Rahman M, Peterson S, Bishai D (2009). Changes in utilization of health services among poor and rural residents in Uganda: are reforms benefitting the poor?. Int J Equity Health.

[27] Ssengooba F, Rahman S, Hongoro C, Rutebemberwa E, Mustafa A, Kielmann T (2007). Health sector reforms and human resources for health in Uganda and Bangladesh: mechanisms of effect. Hum Resour Health.

[28] Kasujja FX, Nuwaha F, Daivadanam M, Kiguli J, Etajak S, Mayega RW (2021). Understanding the diagnostic delays and pathways for diabetes in eastern Uganda: a qualitative study. PLoS ONE.

[29] Inouye SK (2021). Creating an anti-ageist healthcare system to improve care for our current and future selves. Nat Aging.

[30] Aboderin I (2010). Understanding and advancing the health of older populations in sub-saharan Africa: policy perspectives and evidence needs. Public Health Rev.

[31] Yam H-K, Mercer SW, Wong L-Y, Chan W-K, Yeoh E-K (2009). Public and private healthcare services utilization by non-institutional elderly in Hong Kong: is the inverse care law operating?. Health Policy.

[32] Wandera SO, Kwagala B, Ntozi J (2015). Determinants of access to healthcare by older persons in Uganda: a cross-sectional study. Int J Equity Health.

[33] George P, Heng B, De Castro Molina J, Wong L, Wei Lin N, Cheah JT (2012). Self-reported chronic diseases and health status and health service utilization - results from a community health survey in Singapore. Int J Equity Health.

[34] Gómez-Olivé FX, Thorogood M, Clark B, Kahn K, Tollman S (2013). Self-reported health and health care use in an ageing population in the Agincourt sub-district of rural South Africa. Global Health Action.

[35] Channon AA, Andrade MV, Noronha K, Leone T, Dilip TR (2012). Inpatient care of the elderly in Brazil and India: assessing social inequalities. Soc Sci Med.

[36] Luo J, Zhang X, Jin C, Wang D (2009). Inequality of access to health care among the urban elderly in northwestern China. Health Policy.

[37] UN. Madrid International Plan of Action on Ageing (MIPAA) New York (NY). United Nations; 2002.

[38] Mulumba M, Nantaba J, Brolan C, Ruano A, Brooker K, Hammonds R (2014). Perceptions and experiences of access to public healthcare by people with disabilities and older people in Uganda. Int J Equity Health.

[39] Armstrong-Hough M, Sharma S, Kishore SP, Akiteng AR, Schwartz JI (2020). Variation in the availability and cost of essential medicines for non-communicable diseases in Uganda: a descriptive time series analysis. PLoS ONE.

[40] Tusubira AK, Akiteng AR, Nakirya BD, Nalwoga R, Ssinabulya I, Nalwadda CK (2020). Accessing medicines for non-communicable diseases: patients and health care workers’ experiences at public and private health facilities in Uganda. PLoS ONE.

[41] Ng G, Raskin E, Wirtz VJ, Banks KP, Laing RO, Kiragu ZW (2021). Coping with access barriers to non-communicable disease medicines: qualitative patient interviews in eight counties in Kenya. BMC Health Serv Res.

[42] Rogers HE, Akiteng AR, Mutungi G, Ettinger AS, Schwartz JI (2018). Capacity of Ugandan public sector health facilities to prevent and control non-communicable diseases: an assessment based upon WHO-PEN standards. BMC Health Serv Res.

[43] MRC UVRI (2011). Direct and indirect effects of HIV/AIDS and anti-retroviral treatment on the health and wellbeing of older people.

[44] Kuteesa MO, Seeley J, Cumming RG, Negin J (2012). Older people living with HIV in Uganda: understanding their experience and needs. Afr J AIDS Res.

[45] Zakumumpa H, Kiweewa FM, Khuluza F, Kitutu FE (2019). The number of clients is increasing but the supplies are reducing: provider strategies for responding to chronic antiretroviral (ARV) medicines stock-outs in resource-limited settings: a qualitative study from Uganda. BMC Health Serv Res.

[46] Nankwanga A, Phillips J, Neema S (2009). Exploring and curbing the effects of HIV/AIDS on elderly people in Uganda. J Community Health Sci.

[47] Ntozi JPM, Nakayiwa S. AIDS in Uganda: how has the household coped with the epidemic. The continuing African HIV/AIDS epidemic. 1999;2:155– 81.

[48] Seeley J, Kabunga E, Tumwekwase G, Wolff B, Grosskurth H. The Impact of the AIDS Epidemic on the Lives of Older People in Rural Uganda. DEV Working Paper 04; 2008.

[49] Seeley J, Wolff B, Kabunga E, Tumwekwase G, Grosskurth H (2009). This is where we buried our sons’: people of advanced old age coping with the impact of the AIDS epidemic in a resource-poor setting in rural Uganda. Aging Soc.

[50] Golaz V, Rutaremwa G (2011). The vulnerability of older adults: what do census data say? An application to Uganda. Afr Popul Stud.

[51] Golaz V, Rutaremwa G, Wandera SO, Adjamagbo A, Antoine P (2015). Les solidarités familiales autour des personnes âgées en Ouganda. Démographie et politiques sociales (Actes Du XVIIe colloque, Ouagadougou, Novembre 2012). Association Internationale Des Démographes De Langue Française (AIDELF), 2015.

[52] Hjelm K, Atwine F. Health-care seeking behaviour among persons with diabetes in Uganda: an interview study. BMC Int Health Hum Rights. 2011;11(11). 10.1186/1472-698X-11-11 PMC3213135 21943099

[53] Birabwa C, Bwambale MF, Waiswa P, Mayega RW (2019). Quality and barriers of outpatient diabetes care in rural health facilities in Uganda - a mixed methods study. BMC Health Serv Res.

[54] Wandera SO. Disparities in Health and Access to Healthcare Among Older Persons in Uganda [Thesis by Publications ]. Kampala, Uganda: Makerere University. 2016.

[55] MPRAM. Multi-disciplinary Partner Team on Poverty, Resource Accessibility, and spatial Mobility in East Africa: MPRAM. ; 2016. Available from: https://mpramea.wordpress.com/our-projects/bunyoro/.

[56] Gale N, Heath G, Cameron E, Rashid S, Redwood S (2013). Using the framework method for the analysis of qualitative data in multi-disciplinary health research. BMC Med Res Methodol.

[57] Ostlund U, Kidd L, Wengstrom Y, Rowa-Dewar N. Combining qualitative and quantitative research within mixed method research designs: a methodological review. Int J Nurs Stud. 2010. 10.1016/j.ijnurstu.2010.10.005 PMC7094322 21084086

[58] Obrist B, Iteba N, Lengeler C, Makemba A, Mshana C, Nathan R (2007). Access to Health Care in contexts of Livelihood Insecurity: a Framework for Analysis and Action. PLoS Med.

[59] Chen J, Ssennyonjo A, Wabwire-Mangen F, Kim JH, Bell G, Hirschhorn L (2021). Does decentralization of health systems translate into decentralization of authority? A decision space analysis of Ugandan healthcare facilities. Health Policy Plan.

[60] Kiwanuka SN, Ekirapaa EK, Peterson S, Okuia O, Hafizur Rahman M, Peters D (2008). Access to and utilisation of health services for the poor in Uganda: a systematic review of available evidence. Royal Soc Trop Med Hygiene.

[61] Mamdani M, Bangser M (2004). Poor People’s Experiences of Health Services in Tanzania. Reprod Health Matters.

[62] Schäfermann S, Neci R, Ndze EN, Nyaah F, Pondo VB, Heide L (2020). Availability, prices and affordability of selected antibiotics and medicines against non-communicable diseases in western Cameroon and northeast DR Congo. PLoS ONE.

[63] Onyango MA, Vian T, Hirsch I, Salvi DD, Laing R, Rockers PC (2018). Perceptions of Kenyan adults on access to medicines for non-communicable diseases: a qualitative study. PLoS ONE.

[64] Lugada E, Komakech H, Ochola I, Mwebaze S, Olowo Oteba M, Okidi Ladwar D (2022). Health supply chain system in Uganda: current issues, structure, performance, and implications for systems strengthening. J Pharm Policy Pract.

[65] Masters SH, Burstein R, DeCenso B, Moore K, Haakenstad A, Ikilezi G (2014). Pharmaceutical availability across levels of care: evidence from facility surveys in Ghana, Kenya, and Uganda. PLoS ONE.

[66] Okiror B, Onchweri AN, Miruka CO, Maniga JN (2015). Availability of essential Medicines and supplies during the Dual pull-push System of Drugs Acquisition in Kaliro District, Uganda. Pharma Care Health Sys.

[67] Bukuluki P, Byansi PK, Sengendo J, Nyanzi ID, Banoba P, Kaawa-Mafigiri D (2013). Changing from the pull to the Push System of Distributing Essential Medicines and Health Supplies in Uganda: implications for efficient allocation of Medicines and Meeting the Localized Needs of Health Facilities. GLOBAL HEALTH Gov.

[68] Peters DH, Garg A, Bloom G, Walker DG, Brieger WR, Rahman MH (2008). Poverty and access to health care in developing countries. Ann N Y Acad Sci.

[69] McIntyre D, Thiede M, Birch S (2009). Access as a policy-relevant concept in low- and middle-income countries. Health Econ Policy Law.

[70] MoGLSD (2011). Income Security for all ugandans in Old Age.

